# Advantageous factors of R0 curative conversion esophagectomy and the optimal extent of lymphadenectomy after induction therapy for cT4b thoracic esophageal cancer

**DOI:** 10.1002/ags3.12416

**Published:** 2020-12-11

**Authors:** Yu Ohkura, Masaki Ueno, Harushi Udagawa

**Affiliations:** ^1^ Department of Gastroenterological Surgery Toranomon Hospital Okinaka Memorial Institute for Medical Research Tokyo Japan

**Keywords:** advantageous factors, conversion esophagectomy, T4b, thoracic esophageal cancer

## Abstract

**Aim:**

This study aimed to clarify the prognostic factors, the advantageous factors of R0 curative resection, and optimal extents of lymph node dissection for conversion esophagectomy after induction therapy.

**Methods:**

Among 1903 patients with esophageal cancer at Toranomon Hospital between January 2006 to May 2020, 151 patients with locally advanced T4b thoracic esophageal cancer were divided into two groups according to treatment: conversion surgery group (n = 54) and non‐surgical treatment group (n = 97) for comparison.

**Results:**

The patients who underwent R0 curative resection showed preferable survival comparable to the survival rate of patients with cCR in the non‐surgical treatment group (1‐, 3‐ and 5‐year survival: 96.9%, 82.1% and 76.7% vs 94.1%, 86.3%, and 86.3%; *P* = 0.770). Multivariate analysis revealed that the T4b tumor invasion by primary site (odds ratio (OR) = 6.100; 95% CI, 1.439‐25.865: *P* = 0.014) and time to conversion surgery from start of induction therapy within four months (OR = 5.229; 95% CI, 1.296‐21.102: *P* = 0.020) were all independent advantageous factors of R0 curative resection. Actuarial 1‐, 3‐ and 5‐year survival rates in patients who underwent conversion surgery with D2‐3 lymphadenectomy were 90.9%, 48.6%, and 40.8%, respectively.

**Conclusions:**

R0 resection led to improved prognosis in conversion esophagectomy for cT4b esophageal cancer. The T4b tumor invasion by primary site and time to conversion surgery from start of induction therapy within 4 months were independent advantageous factors of R0 curative resection. In addition, standard radical esophagectomy including prophylactic D2‐/3‐ lymphadenectomy should be performed if it is possible, while taking adequate care regarding the increased risk after induction therapy.

## Graphical abstract

In conversion esophagectomy for T4b esophageal cancer after induction therapy, R0 curative resection was found to be the most favorable prognostic factor. The T4b tumor invasion by primary site and time to conversion surgery from start of induction therapy within four months were independent advantageous factors of R0 curative resection. In addition, typical esophagectomy including prophylactic D2 or D3 lymphadenectomy should be performed if it is possible, while taking adequate care to avoid damage to the aorta and trachea.

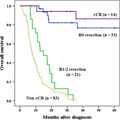

## 1 INTRODUCTION

The standard treatment for stage II and III esophageal cancer is neoadjuvant chemotherapy (NACT) or neoadjuvant chemoradiotherapy (NACRT) + radical surgery. However, definitive chemoradiotherapy (DCRT) with organ preservation is an option for esophageal cancer and has become one of the most common nonsurgical treatments for locally advanced esophageal cancer particularly when difficulty in R0 resection is suspected. In our hospital, we tend to opt for DCRT for patients with unresectable cT4b cancer. However, the complete response (CR) rate of patients with cT4 cancer who received DCRT was about 17%–39%, which is considerably lower than that of T3 cases; 64%–69.2%.^1^, ^2^, ^3^, ^4^ Therefore, if the tumor invasion is relieved by induction chemotherapy or chemoradiotherapy, conversion esophagectomy emerges as one of the radical treatment options. However, a suitable treatment strategy for cT4b cases has not been established and remains unclear. The aim of the present study was to clarify the effectiveness of conversion esophagectomy after induction therapy, the advantageous factors of R0 curative conversion esophagectomy and the treatment strategies such as extent of lymphadenectomy especially for locally advanced T4b esophageal cancer.

## 2 METHODS

### 2.1 Study population

**Figure 1:**
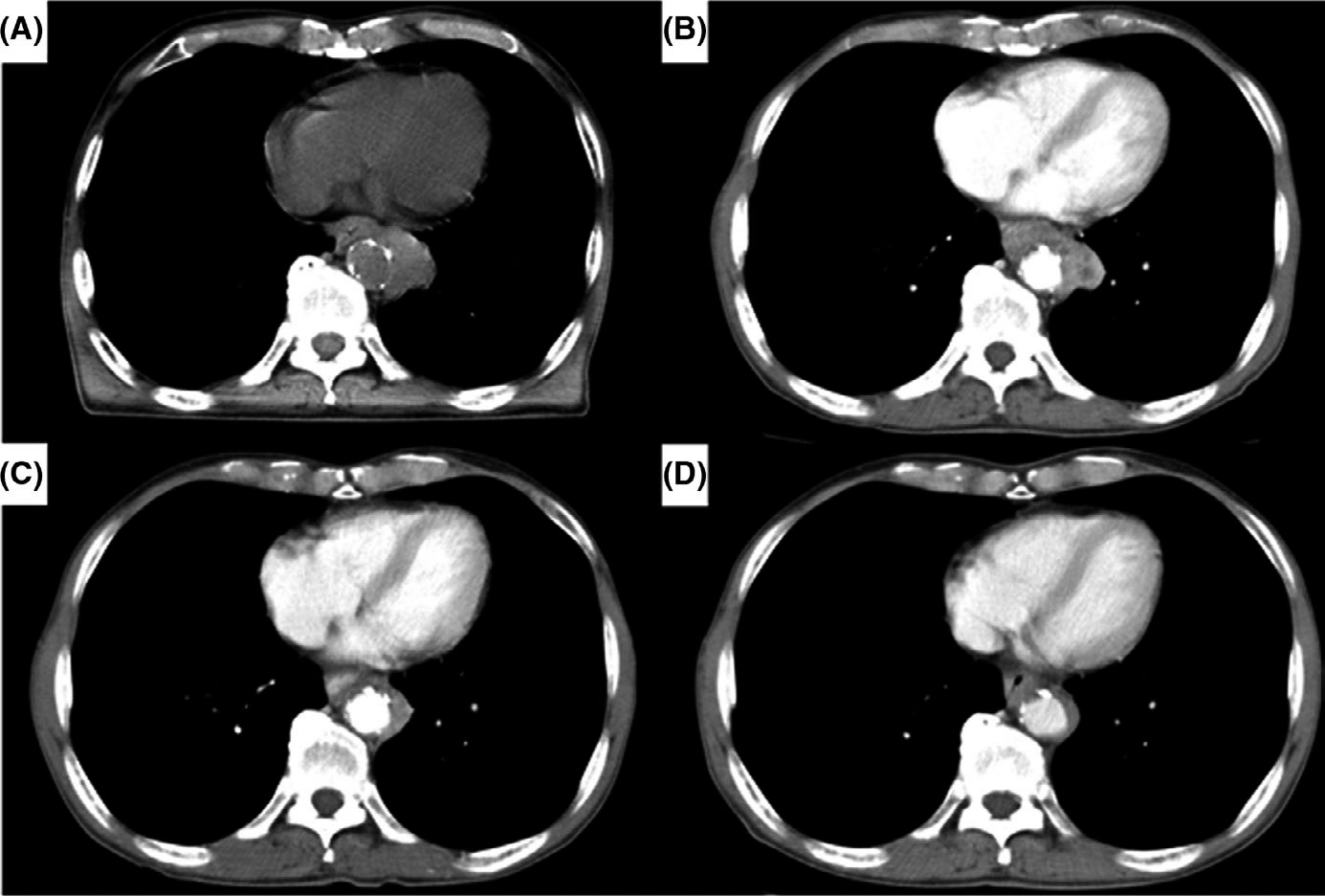
Patient who underwent R0 curative resection after induction therapy (5‐fluorouracil+cisplatin:FP + 40 Gy). A, Primary tumor and metastatic lymph node invading the aorta before induction therapy in non‐contrast‐enhanced computed tomography (CT) scan. B, Primary tumor and metastatic lymph node invading the aorta before induction therapy in contrast‐enhanced CT scan. C, The tumor immediately after induction therapy. D, The tumor after about 4 wks after induction therapy

In this single‐center retrospective study, a total of 1903 consecutive patients with esophageal cancer were identified from a database that was prospectively constructed at Toranomon hospital between January 2006 and May 2020. Among these, 151 patients who had thoracic esophageal cancer invading adjacent vital structures (aorta and/or trachea/bronchus) without distant organ metastases were selected for this study. Patients with cervical lymph node metastases were included. We indicate by “bulky T3,” a T3 tumor which has the potential of direct invasion to adjacent organs but safe resection is highly probable regardless of microscopic radial margin diagnosis. We think it is very difficult to clearly differentiate resectable bulky T3 and real T4 tumors. In this study, we selected only T4b tumors which were clearly diagnosed before treatment. We did not include the patients with bulky T3 tumors. We present an example of a patient with apparent T4b selected in this study in Figure 1. These 151 patients were divided into two groups according to treatment: conversion esophagectomy group (n = 54) and non‐surgical treatment group (n = 97) for comparison. We evaluated the prognostic factors, surgical indications, optimal extent of lymph node dissection and the effectiveness of conversion esophagectomy compared with non‐surgical treatment. Furthermore, we analyzed the advantageous factors of R0 curative resection by comparing the R0 resection group and the R1/2 resection group using univariate and multivariate analysis. We judged whether or not R0 resection had occurred by pathological findings after operation in the conversion esophagectomy group. We investigated the association between these significant factors and the tumor differentiation for the interpretation of these advantageous factors. Assessment of invasion of adjacent structures was performed using enhanced computed tomography (CT) scan, magnetic resonance imaging (MRI), endoscopy, endoscopic ultrasonography (EUS) and bronchoscopy before treatment and before surgery. EUS was an excellent method for evaluating tumor invasion of aorta and trachea/bronchus, but sometimes the endoscopy could not pass the tumor because of stenosis caused by the esophageal tumor. In contrast, enhanced CT scan was applied to all the patients even when the esophageal lumen was stenotic. The overall circumference of contact between the tumor and the aortic wall has been shown to be a useful predictor, with an interface arc greater than 90 degrees, suggesting invasion, as reported by Picus et al.^5^ Bronchoscopy was a useful method for evaluating tumor invasion of the trachea/bronchus. These modalities were used to comprehensively assess the presence or absence of tumor invasion of aorta and/or trachea/bronchus at a multidisciplinary conference (surgeons, gastroenterologists, oncologists, radiologists, and pathologists). Diseases were staged according to the UICC TNM grading system, 7th edition.^6^ Although direct invasion of metastatic lymph nodes to the aorta and/or trachea/bronchus are not clearly included in the UICC TNM system, it was included in this study as cT4b. All postoperative complications were graded based on the Clavien‐Dindo classification (CDc),^7^ and grade ≥ III events were documented as complications. This study was conducted with approval from the Institutional Review Board of Toranomon Hospital (approval number 2026).

### 2.2 Non‐surgical treatment

In the present study, induction therapy included chemoradiotherapy (CRT), chemotherapy (CT) alone, CT followed by CRT (CT‐CRT), and CRT followed by CT (CRT‐CT). The chemotherapy regimens in the induction CRT protocol were either 5‐fluorouracil (5‐FU) 700 mg/m^2^ on days 1‐4 and cisplatin 70 mg/m^2^ on day 1 (FP) or docetaxel 75 mg/m^2^ on day 1, cisplatin 75 mg/m^2^ on day 1, and 5‐fluorouracil 750 mg/m^2^ on days 1‐5 (DCF). In the radiation protocol, fraction of 2 Gy/day was given up to 40‐60 Gy. DCRT was defined as chemotherapy combined with ≥50.4 Gy of radiation to the main tumor and detected metastases, and more than 40 Gy of prophylactic radiation to the regional lymph nodes. Induction CT regimen was either FP (800 mg/m^2^ 5FU on days 1‐5, 80 mg/m^2^ cisplatin on day 1) or DCF (75 mg/m^2^ docetaxel on day 1, 75 mg/m^2^ cisplatin on day 1, 750 mg/m^2^ 5 FU on days 1‐5). Induction CT‐CRT or CRT‐CT protocol was mixed these chemotherapy and chemoradiotherapy protocols mentioned above.

### 2.3 Tumor response

The clinical response was assessed by endoscopy with esophageal biopsy, computed tomography (CT, with PET‐CT in some cases), and ultrasonography of the abdomen and neck. Especially, we diagnosed whether or not there was T4b relief after induction therapy using the same modalities before treatment such as enhanced CT, MRI, bronchoscopic and endoscopic examinations. The clinical response was determined using the RECIST (Response Evaluation Criteria In Solid Tumors) guidelines^8^, ^9^ and the 11th edition of the Japanese Classification of Esophageal Cancer parts II and III.^10^ Responses were classified as follows: CR, PR (partial response), PD, (progressive disease), and SD (stable disease). We selected conversion esophagectomy when the tumor invasion is estimated to be relieved by these induction therapies. Our basic policy for management of patients presenting with cCR is patients’ self‐decision‐making after receiving detailed informed consent regarding the risks and benefits of each treatment option (surveillance or surgery). The cCR for the main lesions was defined as: (a) disappearance of endoscopic findings suggesting presence of a tumor, (b) negative endoscopic biopsy findings from the area of the primary tumor, (c) observation of the entire esophagus is possible using endoscopy, and (d) no endoscopic findings of active esophagitis; the response for lymph node metastases was defined as a reduction in the short axis of the affected lymph nodes to <10 mm on CT and on ultrasonography of the abdomen and neck.

The histopathological effects of induction therapy were defined according to the Japanese Classification of Esophageal Cancer, 11th edition.^10^ The grading systems; Grade 0: Ineffective, no recognizable cytological or histological therapeutic effect. Grade 1: Slightly effective, apparently viable cancer cells account for 1/3 or more of the tumor tissue, but there is some evidence of degeneration of the cancer tissue or cells. Grade 1a: viable cancer cells accounting for 2/3 or more tumor tissue. Grade 1b: viable cancer cells accounting for 1/3 or more, but less than 2/3, of tumor tissue. Grade 2: Moderately effective, viable cancer cells account for less than 1/3 of the tumor tissue, while the other cancer cells are severely degenerated or necrotic. Grade 3: Markedly effective, no viable cancer cells are evident. In this study, multiple pathologists carried out the routine pathology. However, a single chief pathologist reviewed all pathological materials and routinely made a final diagnosis of the histopathological response; namely grade 0 to 3 of induction therapy.

### 2.4 Surgical procedure

We carry out esophagectomy with two‐ or three‐field lymph node dissection depending on the degree of progression and surgical risk involved.^11^ The operative thoracic approach is by video‐assisted thoracoscopic surgery (VATS) or thoracotomy, and the abdominal approach is hand‐assisted laparoscopic surgery (HALS) or open laparotomy depending on individual cases. We generally resected the thoracic duct (TD) in cases with cStage ≥ II for the purpose of lymphadenectomy. However, we try to preserve TD in patients with high risk particularly in hepatic or pulmonary function. We preserved bilateral bronchial arteries to maintain the bronchial blood flow, although they were resected when we suspected tumor invasion to the bronchial artery by primary tumor or metastatic lymph node. A manually sutured esophagogastric or esophago‐ileal anastomosis in the neck was fashioned for all patients. We used three groups of lymph node basins defined in relation to the main tumor location by Japan Esophageal Society to describe the extent of lymph node dissection in an esophagectomy: D0 dissection: no or incomplete dissection of group 1 lymph nodes; D1 dissection: complete dissection of group 1 lymph nodes, but no or incomplete dissection of group 2 lymph nodes; D2 dissection: complete dissection of group 1 and group 2 lymph nodes, but no or incomplete dissection of group 3 lymph nodes; and D3 dissection: complete dissection of groups 1, 2, and 3 lymph nodes.^10^

### 2.5 Determination of treatment policy

In all the T4b cases, we assessed the esophageal cancer itself and the appropriate management of therapy at a multidisciplinary conference (surgeons, gastroenterologists, oncologists, radiologists, and pathologists). Therefore, at this conference, our hospital decided on the chemotherapy regimen, radiation dose, radiation field, and propriety of conversion surgery. Finally, management of therapy was decided by the patient after detailed informed consent was provided regarding the risks and benefits of each treatment option.

### 2.6 Statistics

Cumulative rates of overall survival (OS) were analyzed by the Kaplan‐Meier method. Prognostic factors involved in OS were evaluated using the log‐rank test. In multivariate analysis, variables associated with OS were identified using stepwise Cox proportional hazards models. Variables identified using simple Cox proportional hazards models were selected for potential association with survival based on our clinical experience. Variables with significance of *P* < 0.05 in the simple Cox proportional hazards models were included in multifactorial Cox proportional hazard models. In multiple Cox hazards models, *P* < 0.05 was considered significant. All statistical analyses were carried out using Statistical Package for the Social Sciences (SPSS) version 19.0J for Windows (SPSS Inc., Chicago, IL, USA).

## 3 RESULTS

### 3.1 Patient characteristics

Baseline characteristics of the 151 cases of locally advanced T4b esophageal cancer are shown in Table 1. Of these 151 cases, 54 patients underwent conversion surgery, and 97 patients underwent non‐surgical treatment: 14 patients had cCR and 83 patients had non‐cCR (PR/SD/PD). Of these 54 cases, R0 curative resection was achieved in 33 patients (61.1%), and 21 patients underwent R1/2 non‐curative resection. The adjacent structures invaded by the tumor were trachea/bronchus in 96 patients, aorta in 38 patients, and both (trachea/bronchus plus aorta) in 17 patients. Some patients underwent conversion surgery after aortic stent placement for suspicious direct aortic tumor invasion; however, in the present study, there were no patients who required a combined resection of T4b organ invaded by tumor. The cause of T4b invasion was the primary tumor in 113 patients and metastatic lymph node in 38 patients. Clinical stage before treatment was stage IIIC in 99 patients and stage IV in 52 patients. T4b organs, tumor location and clinical stage were significantly different between the two groups. T4b by metastatic lymph node invasion was significantly more frequent in patients with conversion surgery than in patients with non‐surgical treatment. Cases with cervical lymph node metastases were significantly higher in the non‐surgical treatment group than in the conversion surgery group.

**Table 1:** Baseline characteristics of the 209 cases of locally advanced T4b esophageal cancer

	Total (n = 151)	Conversion (n = 54)	Non‐surgery (n = 97)	*P*‐value
Age (mean ± SD)	64.7 ± 9.7	64.3 ± 9.7	64.9 ± 10.0	0.410
Gender (Male/Female)	126/25	43/11	83/14	0.347
Tumor location				
Ut	63	32	31	0.005
Mt	71	17	54	
Lt	17	5	12	
T4b organs				
Trachea/Bronchus	96	39 (72.2%)	57 (58.8%)	0.071
Aorta	38	13 (24.1%)	25 (25.8%)	
Trachea/Bronchus + Aorta	17	2 (3.7%)	15 (15.5%)	
T4b reason				
Primary tumor	113	34 (63.0%)	79 (81.4%)	0.012
Lymph node	38	20 (37.0%)	18 (18.6%)	
Clinical N factor				
cN0	22	6	8	0.767
cN1	76	21	39	
cN2	70	20	32	
cN3	41	7	18	
Clinical M factor				
cM0	99	44	55	0.002
cM1 cervical lymph	52	10	42	
Clinical stage				
IIIC	99	44 (81.5)	55 (56.7)	0.002
IV	52	10 (18.5)	42 (43.3)	
Clinical response				
CR	17	3	14	<0.001
PR	42	30	12	
SD	36	21	15	
PD	56	0	56	

Abbreviations: CR, complete response; Lt, lower thoracic; Mt, middle thoracic; PD, progressive disease; PR, partial response; SD, stable disease; Ut, upper thoracic.

### 3.2 Long‐term prognosis of T4b esophageal cancer

**Figure 2:**
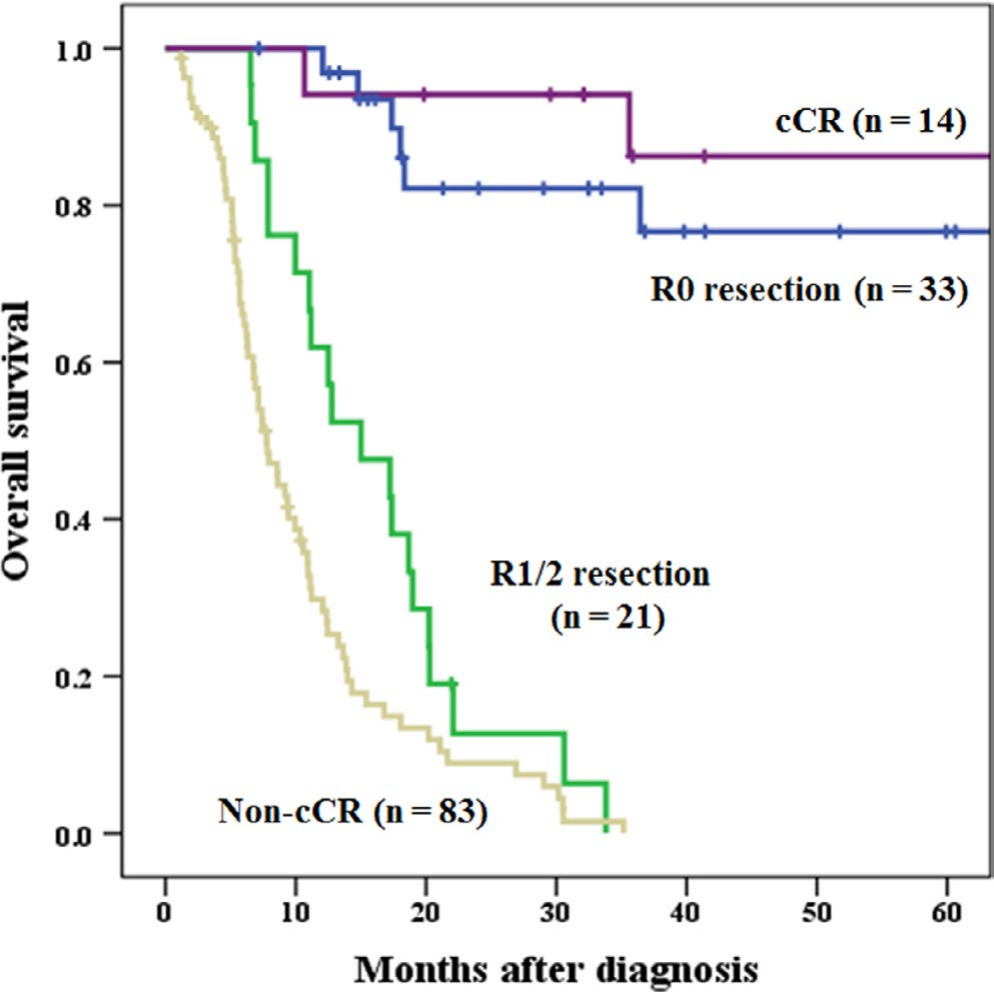
Survival curves for patients with T4b esophageal cancer who underwent R0, R1/2 conversion surgery compared with the non‐surgical group; cCR/non‐cCR

Median observation period for all cases was 95.1 months (Kaplan‐Meier estimate). Actuarial 1‐, 3‐ and 5‐year survival rates were significantly higher in patients with T4b esophageal cancer who underwent conversion surgery (85.0%, 47.3%, and 44.2%, respectively) than in patients who did not (42.0%, 16.3%, and 16.3%, respectively) (data not shown). In subgroup analysis of the conversion surgery group, the survival rates of patients who underwent R0 curative resection were significantly higher than those of patients who underwent R1/2 non‐curative resection (1‐, 3‐ and 5‐year survival: 96.9%, 82.1% and 76.7% vs 61.9%, 0% and 0%, respectively; *P* < 0.001). In subgroup analysis of the non‐surgical treatment group, the survival rates of patients with cCR were significantly higher than those of patients with non‐cCR (1‐, 3‐ and 5‐year survival: 94.1%, 86.3%, and 86.3% vs 29.8%, 0% and 0%, respectively; *P* < 0.001). Patients who underwent R0 curative resection showed survival comparable to patients who did not undergo surgery with cCR (*P* = 0.770) (Figure 2). In contrast, if we could not perform R0 curative resection in the conversion surgery group after induction therapy, the survival rate did not differ significantly between the non‐R0 resection in conversion surgery group and the non‐CR in non‐surgical treatment group (Figure 2). The survival rate of patients who did not undergo R0 resection was significantly higher than that of patients with non‐cCR in the non‐surgical treatment group (*P* = 0.013). Among 54 patients in the conversion surgery group, only three patients had pathological CR in this study. The non‐pCR patients among R0 curative resections (n = 30) showed survival comparable to patients who did not undergo surgery with cCR (n = 14). The survival rate of these 30 R0 but non‐pCR patients was significantly higher than that of patients with non‐cCR (n = 83) in the non‐surgical treatment group (1‐, 3‐ and 5‐year survival: 96.6%, 80.0% and 73.3% vs 29.8%, 0% and 0%, respectively; *P* < 0.001) (data not shown).

#### 3.2.1 Advantageous factors of R0 curative resection of T4b cancer

**Figure 3:**
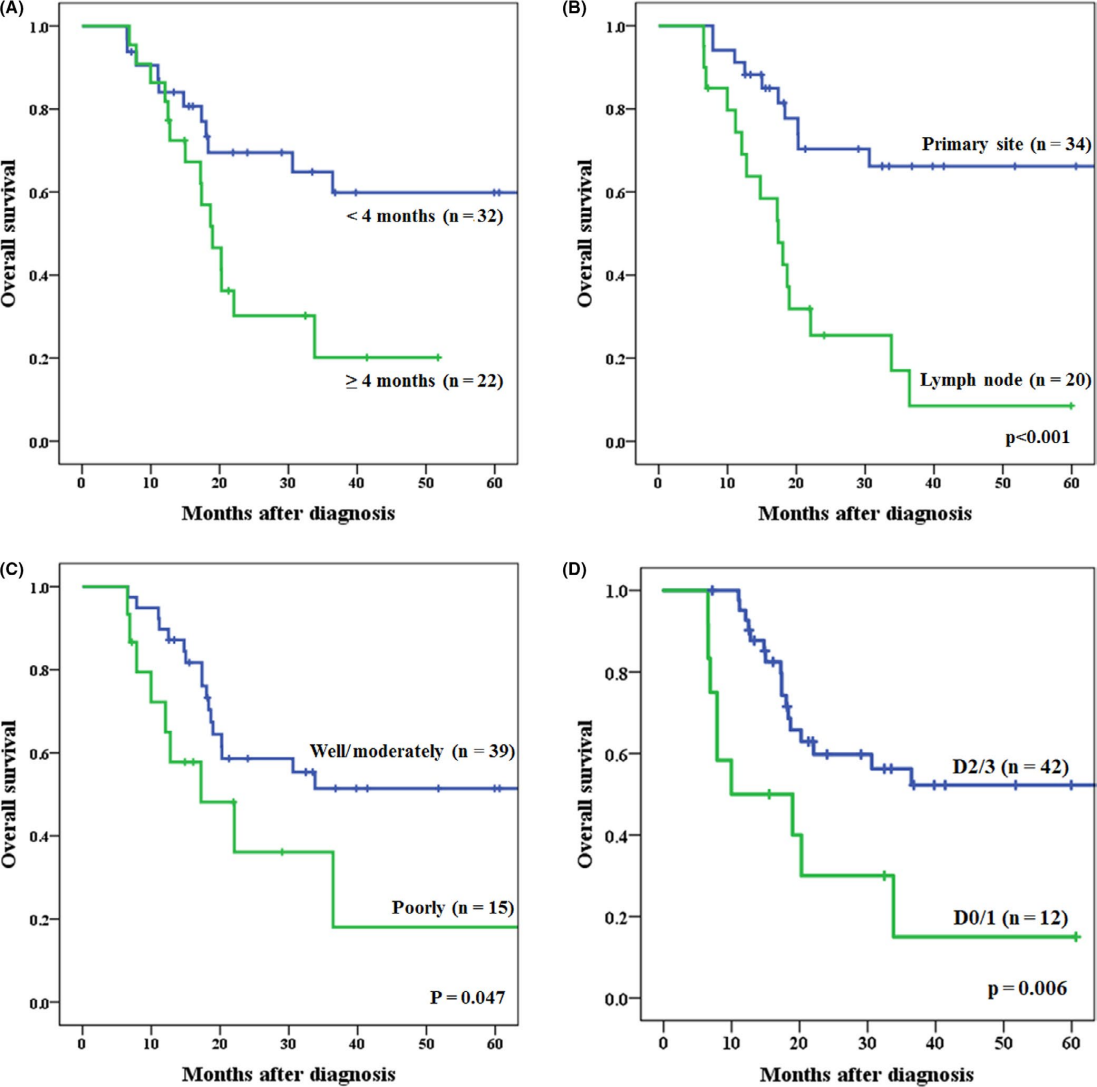
A, Survival curves for patients who underwent conversion surgery within 4 mo from start of induction therapy compared with more than 4 mo. The 1‐, 3‐, 5‐year survival rates; <4 mo: 84.1%, 64.9%, and 59.9% vs ≧4 mo: 86.4%, 20.1% and 20.1% (*P* = 0.017). B, Survival curves for patients with T4b tumor invasion by primary site compared with invasion by metastatic lymph node. The 1‐, 3‐, 5‐year survival rates; primary site: 88.2%, 66.2%, and 66.2% vs lymph node: 74.4%, 17.0% and 8.5% (*P* < 0.001). C, Survival curves for patients with poorly differentiated carcinoma compared with well/moderately differentiated carcinoma. The 1‐, 3‐, 5‐year survival rates; poorly: 72.2%, 36.1%, and 18.1% vs well/moderately: 89.7%, 51.4% and 51.4% *P* = 0.047. D, Survival curves for patients with D2‐/D3‐lymphadenectomy compared with D0/1 lymphadenectomy. The 1‐, 3‐, 5‐year survival rates; D2/3 lymphadenectomy: 95.1%, 56.3%, and 52.2% vs D0/1 lymphadenectomy: 50.0%, 15.0% and 15.0% (*P* = 0.006)

Baseline characteristics of 54 cases who underwent conversion surgery are shown in Table 2. The advantageous factors in predicting R0 curative resection are shown in Table 2. Univariate analysis showed significant differences in four factors: T4b reasons (primary tumor/lymph node), time to conversion surgery from start of induction therapy (<4 months/≥4 months), thoracic approach (open/VATS), and extent of lymphadenectomy (D2‐3/D0‐1). Three of these four factors, ie, T4b reason, time to conversion surgery from start of induction therapy, thoracic approach, and two other variables of age and gender were entered into multivariate analysis. We excluded the variables of extent of lymphadenectomy because of the possibility of the largest selection bias that the intraoperative decision to perform extended lymph node dissection should have been largely affected by intraoperative judgement of curative status of surgical margin. Multivariate analysis showed that T4b tumor invasion by primary site (OR = 6.100; 95% CI, 1.439‐25.865: *P* = 0.014) and time to conversion surgery from start of induction therapy was within 4 months (OR = 5.229; 95% CI, 1.296‐21.102: *P* = 0.020) were all independent advantageous factors of R0 curative resection. However, the thoracic approach (open thoracotomy) was not a significant independent advantageous factor of R0 curative resection (OR = 4.167; 95% CI: 0.811‐21.414, *P* = 0.087) (Table 3). Survival curves for patients who underwent conversion surgery within 4 months from start of induction therapy compared with those who were operated later than 4 months are shown in Figure 3A. The survival rate of patients who underwent conversion surgery within 4 months from start of induction therapy was significantly higher than that of patients who underwent conversion surgery later than 4 months (*P* = 0.017). Survival curves for patients with the T4b tumor invasion by primary site compared with invasion by metastatic lymph node are shown in Figure 3B. The survival rate of the patients with T4b tumor invasion by primary site was significantly higher than that of patients with invasion by metastatic lymph node (*P* < 0.001). Table 4 shows the relationship between the origin of tumor invasion and histological type. The rate of poorly differentiated tumor was significantly higher in patients with invasion by metastatic lymph node as T4b reasons than that of primary tumor invasion (*P* = 0.005). In the present study, the survival rate of patients with poorly differentiated tumor was significantly lower than that of patients with well/moderately differentiated tumor (*P* = 0.047; Figure 3C).

**Table 2:** Results of univariate analysis of the factors of R0 curative resection of locally advanced T4b esophageal cancer

	Total (n = 54)	R0 resection (n = 33)	R1/2 resection (n = 21)	*P*‐value
Age (mean ± SD)	64.3 ± 9.2	65.3 ± 9.1	62.7 ± 9.2	0.666
Gender (Male/Female)	43/ 11	25/ 8	18/ 3	0.376
BMI	20.7 ± 2.8	20.5 ± 2.8	20.9 ± 2.8	0.393
T4b organs				
Trachea/Bronchus	39	21	18	0.173
Aorta	13	10	3	
Trachea/Bronchus + Aorta	2	2	0	
T4b reason				
Primary tumor	34	25	9	0.015
Lymph node	20	8	12	
Tumor location				
Ut/ Mt/ Lt	32/ 17/ 5	19/ 9/ 5	13/ 8/ 0	0.157
Clinical N factor				
cN0/ cN1/ cN2/ cN3	6/ 21/ 20/ 7	4/ 12/ 14/ 3	2/ 9/ 6/ 4	0.601
Clinical M factor				
cM0/ cM1 cervical lymph	44/ 10	29/ 4	15/ 6	0.129
Induction therapy				
CRT/ CT/ CT‐CRT/ CRT‐CT	37/ 8/ 7/ 2	23/ 7/ 3/ 0	14/ 1/ 4/ 2	0.090
Radiation dose				
0 Gy: CT	8	7	1	0.117
<50 Gy: CRT/CT‐CRT/CRT‐CT	16:16/ 0/ 0	11:11/ 0/ 0	5:5/ 0/ 0	
≥50 Gy: CRT/CT‐CRT/CRT‐CT	30:21/ 7/ 2	15:12/ 3/ 0	15:9 /4/ 2	
Time to conversion surgery from start of induction therapy				
<4 mo/≥4 mo	32/ 22	25/ 8	7/ 14	0.002
Clinical response				
CR	3	3	0	0.356
PR	30	18	12	
SD	21	12	9	
PD	0	0	0	
Thoracic approach				
Open/VATS	21/ 37	15/ 18	3/ 18	0.018
Abdominal approach				
HALS/ Open/ Lap	30/ 22/ 2	21/ 10/ 2	9/ 12/ 0	0.103
Thoracic duct				
Resection/ Preserve	43/ 11	28/ 5	15/ 6	0.233
Reconstruction organs				
Gastric/ Ileocolon/ Other	45/ 7/ 2	26/ 5/ 2	19/ 2/ 0	0.401
Reconstruction route				
Retrosternal	42	24	18	0.390
Posterior mediastinal	10	8	2	
Ante‐thoracic	2	1	1	
Lymphadenectomy				
D2‐3/D0‐1	42/ 12	30/ 3	12/ 9	0.004
Efficacy of induction therapy				
Grade 0‐1	43	22	17	0.253
Grade 2‐3	15	11	4	
Histological type				
Well/moderately differentiated	39	26	13	0.177
Poorly differentiated	15	7	8	
Pathological T factor				
T0/ T1b/ T2/ T3/ T4a/ T4b	4/ 2/ 4/ 29/ 3/ 12	3 /2 / 4/ 18/ 3/ 3	1/ 0/ 0/ 11/ 0/ 9	0.027
Pathological N factor				
N0/ N1/ N2/ N3	23/ 12/ 13/ 6	15/ 5/ 9/ 4	8/ 7/ 4/ 2	0.473
Pathological stage				
0/ IA/ IB/ IIA/ IIB	3/ 0/ 2/ 9/ 1	3/ 0/ 2/ 5/ 0	0/ 0/ 0/ 4/ 1	0.376
IIIA/ IIIB/ IIIC/ IV	9/ 8/ 12/ 10	8/ 5/ 5/ 5	1/ 3/ 7/ 5	

Abbreviations: BMI, body mass index; CR, complete response; CRT, chemoradiotherapy; CRT‐CT, CRT followed by CT; CT, computed tomography; CT‐CRT, CT followed by CRT; HALS, hand‐assisted laparoscopic surgery; Lt, lower thoracic; Mt, middle thoracic; PD, progressive disease; PR, partial response; SD, stable disease; Ut, upper thoracic; VATS, video‐assisted thoracoscopic surgery .

**Table 3:** Results of multivariate analysis of the advantageous factors of R0 curative resection of locally advanced T4b esophageal cancer

	Odds ratio	95% CI	*P*‐value
T4b tumor invasion by primary site	6.100	1.439‐25.865	0.014
Time to conversion surgery from start of induction therapy: <4 mo	5.229	1.296‐21.102	0.020
Thoracic approach: Open thoracotomy	4.167	0.811‐21.414	0.087

**Table 4:** Relationship between the reasons for tumor invasion and histological type

	T4b reasons	
	Primary site (n = 34)	Lymph node (n = 20)
Histological type (*P* = 0.005)		
Well/moderately differentiated	29	10
Poorly differentiated	5	10

#### 3.2.2 Extent of lymphadenectomy of conversion surgery

**Figure 4:**
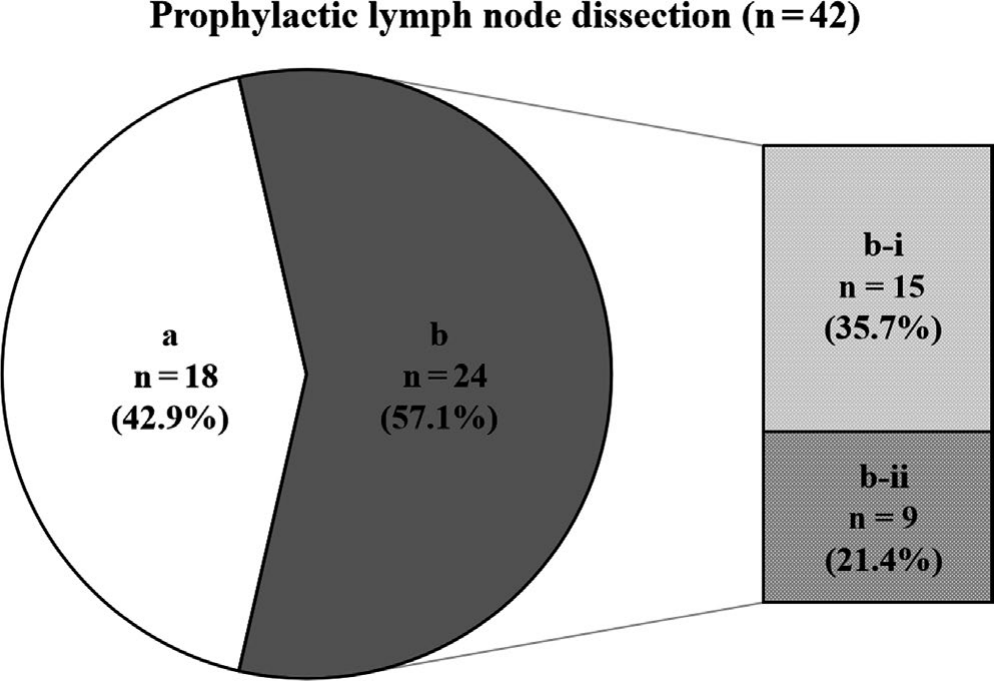
The 42 patients who underwent two‐ or three‐field lymph node dissection including prophylactic dissection. A, Regional lymph node metastasis (–). B, Regional lymph node metastasis (+). B‐i, Neither enlargement nor suspicion before conversion surgery. B‐ii, Suspicion of metastasis before conversion surgery

At our institution, of the 54 locally advanced T4b esophageal cancer patients who underwent conversion surgery, 42 (77.8%) patients underwent D2/3 lymphadenectomy including prophylactic dissection, similar to our standard esophageal cancer surgery. Survival curves for patients who underwent D2/3 lymphadenectomy compared with those who underwent D0/1 lymphadenectomy are shown in Figure 3D. Actuarial 1‐, 3‐ and 5‐year survival rates in patients who underwent conversion surgery with D2‐3 lymphadenectomy were 90.9%, 48.6%, and 40.8%, respectively. The survival rate of patients who underwent D2/3 lymphadenectomy was significantly higher than that of patients who underwent D0/1 lymphadenectomy (*P* = 0.006). Of these 42 patients who underwent prophylactic lymph node dissection, 24 patients (57.1%) had histopathologically proven cancer cells within the dissected regional lymph nodes. Among 24 patients, 15 patients (35.7%) had regional lymph node metastases that had neither enlargement nor suspicious findings before induction therapy and before conversion surgery (Figure 4).

## 4 DISCUSSION

The present study describes the effectiveness of conversion esophagectomy at our institution, evaluating the advantageous factors of R0 curative resection and optimal extent of lymph node dissection in conversion esophagectomy after induction therapy in locally advanced T4b esophageal cancer. The study showed that in conversion esophagectomy for T4b esophageal cancer after induction therapy, R0 curative resection showed better survival comparable to the survival rate of patients with cCR (non‐surgical group). T4b tumor invasion by primary site and time to conversion surgery from start of induction therapy within 4 months were all independent advantageous factors of R0 curative resection. Moreover, regarding the extent of lymphadenectomy, 2‐ or 3‐field lymph node dissection, including prophylactic lymph node dissection is desirable.

Previous studies have reported that conversion surgery after induction therapy can be a potentially curative treatment option for selected patients with cT4b esophageal cancer.^12^ The present study provided a similar result in that the patients who underwent R0 curative resection showed better survival comparable to the survival rate of patients with cCR. However, the optimal extent of lymph node dissection of conversion esophagectomy and the advantageous factors of R0 curative resection after induction therapy in locally advanced T4b esophageal cancer have been, as yet, unclear. Consequently, the strategy for a cT4b esophageal tumor showing good response to induction therapy varies by individual case and by institution and, at present, lacks a consistent approach. We therefore showed the current state of conversion esophagectomy after induction therapy of locally advanced T4b esophageal cancer.

We defined salvage esophagectomy as the operation for residual or relapsed esophageal cancer after DCRT. In contrast, we defined conversion esophagectomy as the operation when T4b tumor invasion is relieved by induction chemotherapy or chemoradiotherapy. If a patient with initially T4b tumor underwent definitive chemoradiotherapy and CR was not obtained but downstaged to a resectable level, and resection was done, such a patient can be a subject of both studies. In our previous report,^13^ among the 57 patients, the number of patients with T4b esophageal cancer was only 22 patients. These patients received only DCRT before conversion surgery, and did not receive chemotherapy before or after DCRT. Of these 22 patients, 20 patients had thoracic esophageal cancer. Among these 20 patients, 14 patients had residual tumors and 6 had tumor relapse after DCRT for esophageal cancer. Therefore, 14 patients were selected both in previous salvage study and in present conversion study. In this study, among 37 patients who received only chemoradiotherapy as induction therapy, 21 patients were treated by ≥50 Gy radiation. Of these 21 patients, 14 patients, ie the same 14 patients mentioned above, were selected both in a previous salvage study and in the present conversion study.^13^ The other 7 patients are new cases in 2018‐2020, and are not included in our previous report.^13^

R0 curative resection in conversion esophagectomy leads to improved prognosis. However, whether or not R0 resection is possible is often difficult to be determined preoperatively using current imaging techniques in original cT4b cases.^13^, ^14^ There were no reports that clarified the advantageous factors of R0 curative resection in the past. In the present study, T4b tumor invasion by primary site compared with lymph node metastasis and time to conversion surgery from the start of induction therapy within 4 months were independent advantageous factors of R0 curative resection. We guessed that these two factors were associated with treatment sensitivity of the tumor.

Because DCRT needs 1 and half months, and another 1 month is necessary for judgement of response and patients’ recovery from the adverse effects, a 3‐month interval from the start of induction therapy and surgery is minimal. Therefore, “time to conversion surgery from start of induction therapy within 4 months” means that the tumor showed good response to the treatment and R0 curative resection was highly expected.

Similar to our result, in univariate analysis, Miyata et al^12^ reported that the survival rate of patients with T4b tumor invasion by primary site tend to be higher than that of patients with invasion by metastatic lymph node (*P* = 0.067). A possible explanation for this is that there are differences in tumor characteristics between these two groups. Although histological type was not an independent advantageous factor of R0 curative resection in the current study, the rate of poorly differentiated tumor was significantly higher in patients with T4b invasion by metastatic lymph node than that by primary tumor invasion (*P* = 0.005), as is shown in Table 3. Poorly differentiated tumors would have high risk of invasion and strong treatment resistance. The high malignant potential of the tumor associated with treatment resistance may lead to non‐curative resection.

In the present study, one of the advantageous factors in predicting R0 curative resection was open thoracotomy as thoracic approach in univariate analysis, although this factor was not a significant independent advantageous factor in multivariate analysis (OR = 4.167; 95% CI: 0.811‐21.414, *P* = 0.087). Thoracoscopic (VATS) approach offers better visualizations of anatomical structures more clearly because of the magnification. However, we believe that tactile sensation is equally or more important than visual perception, especially in T4b esophageal cancer surgery. We may assume that the number of VATS surgeries has been increasing, which led to fewer R0 surgeries because of the lack of tactile sensation compared to open thoracotomy, although we cannot clearly conclude this relationship. We may also state that open thoracotomy used to be the standard approach, and that the indication for conversion surgery was more strictly controlled. In other words, the indication for conversion surgery may not be strictly controlled in recent years when the minimally invasive surgery spread. There is the belief that we should change to a bypass operation when we cannot remove it surgically because of its minimal invasiveness; this is because the hurdle to a conversion surgery decreased. Thus, there are more cases with R0 curative resection with open thoracotomy.

Conversion esophagectomy after induction therapy especially DCRT is considered technically difficult, since the tissues are hardened from the fibrosis due to radiation therapy and have been reported to have a higher postoperative complication rate compared with regular esophageal cancer surgery.^13^, ^14^, ^15^, ^16^, ^17^ In order to reduce the rate of postoperative complication, surgeons in many institutions perform primary tumor resection without lymph node dissection, or only dissect enlarged lymph nodes or those suspicious for metastasis.^18^ Miyata et al^12^ reported that 3‐ and 5‐year survival rates in patients who underwent conversion surgery with D2‐3 lymphadenectomy including R0 (n = 64:88.9%) and R1/2 (n = 8:11.1%) resection were 50.4% and 43.1%, respectively. In the present study, actual 1‐, 3‐ and 5‐year survival rates in patients who underwent conversion surgery with D2‐3 lymphadenectomy including R0 (n = 30:71.4%) and R1/2 (n = 12:28.6%) resection were 95.1%, 56.3%, and 52.2%, respectively. These patients in our study showed preferable survival comparable to the past report despite some differences in the R0 resection rate. Also, the survival rate of patients who underwent D2/3 lymphadenectomy was significantly higher than that of patients who underwent D0/1 lymphadenectomy (*P* = 0.006). Furthermore, of our 42 patients who underwent prophylactic lymph node dissection, 24 patients (57.1%) had histopathologically proven viable cancer cells within the dissected regional lymph nodes. Among these 24 patients, 15 patients (35.7%) had regional lymph node metastases that had neither enlargement nor suspicious findings before induction therapy and before conversion surgery. Prophylactic lymph node dissection might have prevented postoperative lymph node recurrence for these 15 patients. We believe that except for cases of apparent non‐curative resection, standard radical lymph node dissection including prophylactic dissection should be attempted, while taking adequate care to prevent postoperative complications.

In this study, only three patients (3/54; 5.6%) had cCR in the conversion surgery group. Among three patients in this study, two patients are still alive and one patient died of aspiration pneumonia.　There is no consensus on the best treatment for patients with resectable esophageal cancer showing cCR to neoadjuvant therapy. “Watch and Wait strategy” after induction therapy for patients with rectal cancer following a clinical complete response (cCR) to neoadjuvant therapy is a nonstandard approach, but it has become more widely practiced with the advent of total neoadjuvant therapy and with increasing demand by patients in the context of a cCR.^19^ However, we retrospectively analyzed the prognosis of esophageal cancer patients who achieved cCR after neoadjuvant therapy by making a comparison between the esophagectomy group and the non‐surgical group.^20^ These findings show that recurrernce‐free survival and disease‐specific survival were significantly better in patients who underwent esophagectomy than in patients who received nonsurgical treatment. Conversely, OS did not differ significantly between the two groups because of the higher risk of late effects (eg, respiratory complications after NACRT such as aspiration pneumonia/respiratory failure).^20^ Further advances in the diagnostic accuracy of treatment modalities have increased the possibility of making a definitive diagnosis of cCR, with the choice of “Watch and Wait strategy” as a viable option.

The largest bias of this study is that the intraoperative decision to perform extended lymph node dissection should have been largely affected by intraoperative judgement of the curative status of the surgical margin. Our study has a single‐center retrospective design and therefore there is a large selection bias from the stage of decision‐making of treatment options, although the present data are based on a prospectively collated database for consecutive patients over a relatively short period. An external validation prospective study involving a sufficient number of patients would be needed to confirm our observations; a multicenter study with a larger number of cases is also warranted.

## 5 CONCLUSIONS

In conversion esophagectomy for T4b esophageal cancer after induction therapy, R0 curative resection was found to be a favorable prognostic factor. The T4b tumor invasion by primary site and time to conversion surgery from the start of induction therapy within 4 months were all independent advantageous factors of R0 curative resection. In addition, standard esophagectomy including prophylactic 2‐ or 3‐field lymphadenectomy should be performed if it is possible, while taking adequate care to prevent postoperative complications and to avoid damage to the aorta and trachea. An important issue for further research is to establish a method for more accurately diagnosing tumor resectability after induction therapy for cT4b esophageal cancer.

## DISCLOSURE

Conflicts of Interest: None declared.

Author Contributions: Yu Ohkura, Masaki Ueno, and Harushi Udagawa designed the study, wrote the manuscript, revised it critically for important intellectual content, and gave final approval of the content. Yu Ohkura, Masaki Ueno and Harushi Udagawa created study materials or recruited patients.
